# Drug-induced CYP induction as therapy for tacrolimus intoxication 

**DOI:** 10.5414/CNCS110744

**Published:** 2022-05-23

**Authors:** John M. Hoppe, Alexander Holderied, Ulf Schönermarck, Volker Vielhauer, Hans-Joachim Anders, Michael Fischereder

**Affiliations:** 1 Division of Nephrology, Department of Medicine IV, LMU Hospital, Munich, and; 2 Department of Nephrology and Medical Intensive Care, Charité, Berlin, Germany

**Keywords:** tacrolimus/toxicity, kidney transplantation, enzyme-induction, rifampin, phenytoin

## Abstract

Management of calcineurin inhibitor (CNI) therapy in kidney transplant recipients may be complicated due to polypharmacy. As CNI undergo extensive metabolism by cytochrome-P450 enzymes (CYP), drug-mediated CYP inhibition poses a risk for elevated CNI blood concentrations. Here, we report on 2 kidney transplant recipients treated with tacrolimus who presented with signs of tacrolimus intoxication at admission. Patient A was started on antiviral medication ombitasvir, paritaprevir, ritonavir, and dasabuvir for hepatitis C virus treatment 3 days prior to hospitalization. Patient B was treated with clarithromycin for pneumonia. Both therapies cause drug-mediated CYP inhibition, and both patients displayed highly elevated tacrolimus serum concentrations and acute kidney injury (Table 1). After application of the CYP-inducing agents rifampicin and phenytoin, respectively, tacrolimus levels were rapidly reduced, and renal function recovered. Treating severe CNI intoxication is an infrequent yet emergent condition. These results add to the knowledge of therapeutic drug-induced CYP induction as rescue therapy.

## Introduction 

Outcomes in kidney transplant recipients have been substantially improved by the use of calcineurin inhibitors (CNI) for prevention of acute allograft rejection. In particular, tacrolimus has reduced the incidence of acute rejection, including the steroid-resistant types of rejection [1]. Yet, polypharmacy has become an increasing problem in the management of CNI therapy [2]. Orally administered tacrolimus undergoes extensive first-pass effects via intestinal and hepatic cytochrome-P450 (CYP) 3A4 enzymes [3]. Therefore, tacrolimus is subject to pharmacokinetic drug interactions, in particular drugs involved in CYP metabolism [3]. Typical CYP inhibitors include macrolide antibiotics, azole antimycotics, various antiviral medications, antineoplastic agents like ceritinib and idelalisib, and other less commonly used drugs like mifepristone. Examples for CYP inducers are rifampicin and various anticonvulsants and mood stabilizers, such as phenytoin, carbamazepine, and St. John’s wort. Therefore, tacrolimus in combination with CYP inhibitors poses a risk for elevated CNI blood concentrations and CNI intoxication. In contrast, co-medication with CYP inducers poses a risk for sub-therapeutic tacrolimus levels. Thus, the use of CYP inducers may counterbalance the effect of CYP inhibitors on CYP substrates. To our knowledge, utilizing CYP-inducing agents for the management of CNI toxicity has been reported in 11 publications [4, 5]. Here, we report on 2 kidney transplant recipients treated with tacrolimus and CYP inhibitors who presented with signs of tacrolimus intoxication at admission and received CYP-inducing agents as rescue therapy (Table 1). 

## Case A 

**Figure 1:**
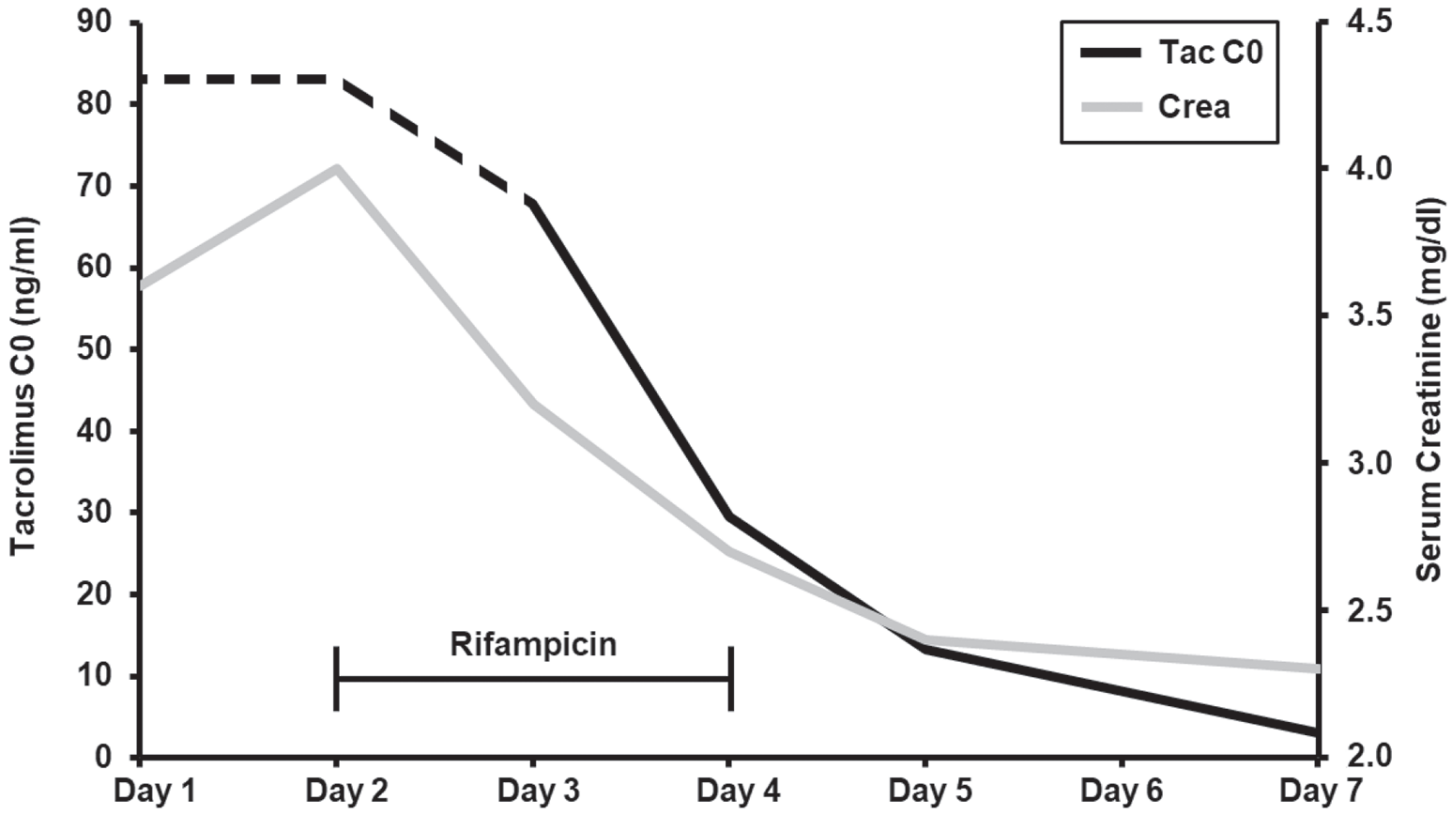
Timeline of tacrolimus trough concentration (black) and serum creatinine (grey) in patient A. Day 1 was day of admission. Dashed line indicates approximate tacrolimus trough concentration, above assay detection limit. Time bar (black) indicates days of rifampicin application.

Patient A is a 56-year-old male kidney transplant recipient (1994) who was transferred to our hospital displaying dyspnea and diffuse abdominal symptoms, including diarrhea and lower abdominal pain. Three days prior to hospitalization, the patient had been started on the antiviral medications ombitasvir, paritaprevir, ritonavir, and dasabuvir for hepatitis C virus treatment. Immunosuppression at admission consisted of tacrolimus, mycophenolate, and low-dose prednisolone. Laboratory tests showed acute chronic kidney injury (creatinine (max) 4.0 mg/dL) and highly elevated tacrolimus trough concentration (tacrolimus C0 (max) 67.8 ng/mL, initially above assay detection limit, ~ 83.0 ng/mL). Chest X-ray detected pulmonary infiltrates, and gastroscopy revealed reflux esophagitis, a florid gastric ulcer, and duodenal erosions, however, there were no signs of helicobacter pylori infection, dysplasia, or malignancy. Furthermore, colonoscopy showed no sign of CMV infection or diverticulitis. Gastric ulceration and pneumonia were treated with proton-pump inhibitors and meropenem, respectively. Tacrolimus and CYP-inhibiting antiviral treatment were paused, and rifampicin (600 mg, 1 – 2×/day for 3 days) was administered as it provides both, antibiotic treatment of gram-positive organisms and CYP induction. Tacrolimus trough levels were monitored frequently. Within the next 5 days, kidney function recovered (creatinine (discharge) 2.1 mg/dL) and tacrolimus concentration returned to therapeutic levels (tacrolimus C0 (discharge) 4.0 ng/mL) (Figure 1). Also, abdominal symptoms subsided. Dyspnea due to pneumonia was treated successfully. 

## Case B 

**Figure 2:**
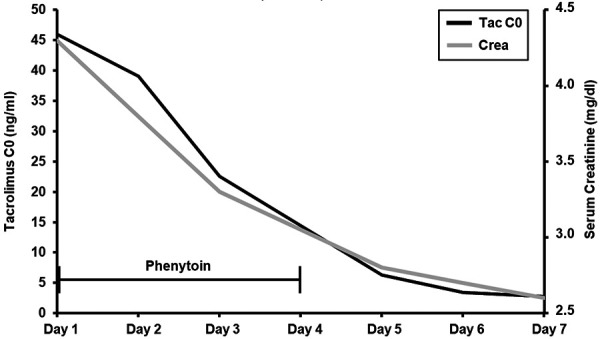
Timeline of tacrolimus trough concentration (black) and serum creatinine (grey) in patient B. Day 1 was day of admission. Time bar (black) indicates days of phenytoin application.

Patient B is a 54-year-old male kidney transplant recipient (1998) who was transferred to our hospital with pneumonia presenting with dyspnea, coughing and fatigue. Pneumonia was treated with clarithromycin. He had a history of multiple cardiac illnesses, including surgical aortic valve replacement, peripheral arterial disease, and coronary heart disease. Further, post-transplant lymphoproliferative disorder with diffuse large B-cell lymphoma was diagnosed in 2012. Immunosuppression at admission consisted of tacrolimus and low-dose cloprednol. Similar to patient A, laboratory testing showed acute chronic kidney injury (creatinine (max) 4.3 mg/dL) and highly elevated tacrolimus trough concentration (tacrolimus C0 (max) 46.0 ng/mL). Tacrolimus was paused, and clarithromycin therapy was switched to piperacillin/tazobactam. Further, the CYP-inducing agent phenytoin (200 mg, 2×/day for 4 days) was started. Within 4 days, tacrolimus levels normalized (tacrolimus C0 (discharge) 4.3 ng/mL), and kidney function recovered (creatinine (discharge) 2.3 mg/dL) (Figure 2). Moreover, pneumonia was treated successfully. 

## Discussion and conclusion 

Tacrolimus toxicity may lead to fatal complications in transplant recipients. Therefore, following diagnosis, rapid correction is advisable. This case report shows a novel therapeutic approach for tacrolimus intoxication in kidney transplant recipients. Here, both the use of rifampicin in patient A and phenytoin in patient B for CYP induction resulted in rapid reduction of tacrolimus trough concentration, subsiding of tacrolimus-dependent symptoms, and full recovery of kidney function. Moreover, there were no signs of adverse reactions to rifampicin or phenytoin, respectively. To mention some possible adverse reactions, rifampicin can cause gastrointestinal symptoms, including heartburn and epigastric distress, transient abnormalities in liver function tests, thrombocytopenia, and nervous system disorders, including headache, fever, and drowsiness [6]. For phenytoin, the most common adverse reactions are central nervous system-related, including nystagmus, ataxia, slurred speech, decreased coordination, somnolence, and mental confusion. Other reactions include suicidal behavior, dermatologic reactions, and hepatic injury. A quick withdrawal may precipitate seizures and status epilepticus [7]. 

Combination therapy with ombitasvir, paritaprevir, ritonavir, and dasabuvir increases the dose-normalized tacrolimus area under the observed concentration curve (AUC) 57-fold in healthy subjects [8]. Consequently, the combination with tacrolimus is contraindicated [9]. The effects of rifampicin on the aforementioned hepaciviral combination drugs have not been tested. However, rifampicin has been reported to decrease the concentration of other antiviral drugs substantially [6]. Also, the CYP inducer carbamazepine has been shown to reduce the AUC of ombitasvir by 31%, paritaprevir by 70, ritonavir by 87%, and dasabuvir by 70% in healthy subjects [9]. Therefore, it can be assumed that rifampicin has similar effects on reducing AUC of the above-mentioned drugs. To our knowledge, there are no reported case reports on the use of rifampicin for CYP induction in CNI-intoxication [4]. Rifampicin is a potent CYP inducer with a half-life of ~ 2 to 3 hours [6] and can lead to subtherapeutic tacrolimus trough levels, even when tacrolimus dosage is significantly increased [10]. Furthermore, the use of rifampicin can provoke acute rejection in transplant recipients under immunosuppressive medication [11]. Studies in human primary hepatocytes have shown rifampicin to induce CYP 3A4 enzymes 55.1-fold [12]. Furthermore, a study, intended to quantify the effects of rifampicin on tacrolimus pharmacokinetics, showed in 6 healthy subjects a significant decrease in tacrolimus oral bioavailability (14 ± 6% vs. 7 ± 3%), a significant increase in tacrolimus clearance (0.036 ± 0.008 vs. 0.053 ± 0.010 L/h/kg), and a decrease in AUC for tacrolimus by 68% with concomitant rifampin administration [13]. For case A, this suggests that rifampicin accelerated tacrolimus elimination via direct CYP induction and via diminished effectiveness of CYP inhibitors due to reduced bioavailability. 

Clarithromycin is a well-described CYP-inhibitor. Serious adverse reactions have been reported in patients taking clarithromycin concomitantly with CYP 3A4 substrates, including colchicine, statins, and calcium channel blockers [14]. Further, a report on 2 female kidney transplant patients, describes a 2.3-fold increase in tacrolimus concentration and acute renal failure after clarithromycin therapy [15]. Similar to rifampicin, phenytoin is a potent CYP inducer. Multiple cases of phenytoin use for CYP induction in CNI intoxication have been described, a review has identified 7 case reports [4]. More recently, a publication has reported on 5 additional cases utilizing phenytoin for CYP induction in CNI intoxication [5]. Furthermore, cases on unintended tacrolimus-phenytoin interactions have been reported. One case describes a 19-year-old female kidney transplant recipient treated with phenytoin for seizures, showing widely varying tacrolimus blood concentrations [16]. In contrast to rifampicin, however, phenytoin is primarily metabolized by CYP enzymes and is particularly susceptible to inhibitory drug interactions [7]. A study in rats showed that clarithromycin increases phenytoin AUC and elimination half-life significantly [17]. On one hand, this may lead to increased phenytoin bioavailability in CYP-inhibited environments, on the other hand, phenytoin elimination kinetics may be difficult to predict, and adverse reactions may arise. A further complicating factor concerning adverse reactions is the relatively long half-life of phenytoin, 22 hours on average (7 – 42 hours) after oral administration [7]. 

A factor that should be taken into account when interpreting the findings for both patients in this case report is CYP inhibition by tacrolimus itself. For instance, the half-life of ciclosporin A is prolonged in patients receiving tacrolimus [18]. Clarithromycin and the hepaciviral drugs, ritonavir, paritaprevir, and dasabuvir, but not ombitasvir, are primarily metabolized via CYP enzymes [9, 14], and therefore increased bioavailability is expected in combination with tacrolimus. This in turn should increase the duration and severity of tacrolimus intoxication. This corresponds well with an increase in elimination half-life, which has been reported to be as high as 41 hours [19]. In our patients, half-lives were calculated using a formula for exponential decay [20]. Half-lives of tacrolimus during CYP induction were similar in both patients, ~ 21.6 hours in patient A and 25.6 hours in patient B, however, estimating the pharmacokinetics of tacrolimus is difficult when at least 3 drug-drug interactions are involved. Interestingly, after CYP induction was terminated, therapeutic tacrolimus levels were maintained with typical tacrolimus dosing in both patients, suggesting short and therefore predictable CYP-inducing effects of rifampicin and phenytoin. 

This case report is limited by the absence of data on patient-specific CYP 3A4-genotyping and patient-specific tacrolimus pharmacokinetics. And since CYP induction has been described as a dynamic process, taking up to 1 – 2 weeks for maximum effect [21], it remains unclear whether tacrolimus concentrations would have normalized at the same rate without administering a CYP-inducing agent. 

A sound risk/benefit assessment should precede any off-label use of pharmaceuticals. Here, we believe the benefits outweighed the risks due to symptomatic tacrolimus intoxication and kidney transplant dysfunction. It is difficult to make recommendations for pharmaco-enhancement with CYP-inducing agents, since this approach will remain experimental. Potent CYP induction, short half-life, and CYP-independent elimination supports the use of rifampicin, particularly in patients showing signs of severe tacrolimus intoxication, including organ dysfunction. Phenytoin, however, may be of advantage in patients presenting with severe neurological symptoms caused by tacrolimus toxicity, such as convulsions and seizures. 

## Funding 

This research received no specific grant from any funding agency in the public, commercial, or not-for-profit sectors. 

## Conflict of interest 

The authors have nothing to disclose. 

**Table 1.** Patient characteristics including age, kidney function, and tacrolimus trough levels at maximum and at discharge, time to normalization of tacrolimus levels, toxicity-inducing drug interaction, and CYP-inducing management strategy.

Identification	Patient A	Patient B
Age (years)	56	54
Toxicity due to interaction with	Ombitasvir, paritaprevir, ritonavir, dasabuvir	Clarithromycin
Tacrolimus C0 (max) (ng/mL)	67.8	46.0
CYP-induction with	Rifampicin, 600 mg, 1 – 2×/day for 3 days	Phenytoin, 200 mg, 2×/day for 4 days
Time to normalization of tacrolimus levels (days)	5	4
Tacrolimus C0 (discharge) (ng/mL)	4.0	4.4
Creatinine (max) (mg/dL)	4.0	4.3
Creatinine (discharge) (mg/dL)	2.1	2.3
